# Editorial Comment: An Effective Evidence-Based Cleaning Method for the Safe Reuse of Intermittent Urinary Catheters: In Vitro Testing

**DOI:** 10.1590/S1677-5538.IBJU.2020.05.04

**Published:** 2020-07-31

**Authors:** Marcio Augusto Averbeck, Blayne Welk

**Affiliations:** 1 Hospital Moinhos de Vento Unidade de Videourodinâmica Porto Alegre RS Brasil Chefe de Neuro-Urologia, Unidade de Videourodinâmica, Hospital Moinhos de Vento. Porto Alegre, RS, Brasil; 2 Western University Department of Surgery and Epidemiology & Biostatistics London ON Canada Department of Surgery and Epidemiology & Biostatistics, Western University, London ON Canada

Wilks SA ^1^^,^^2^, Morris NS ^3^, Thompson R ^3^, Prieto JA ^1^, Macaulay M ^1^, Moore KN ^4^, et al.

## COMMENT

The article published by Wilks et al. (1) is a nice contemporary examination of the in vitro performance of different types of cleaning methods for uncoated urinary catheters. Their methods advance the work of previous studies as they used two types of artificial urine media, assessed for viable but nonculturable bacteria (bacteria which are just “stunned” but could still potentially cause an infection), and assessed surface structural damage. While many of the traditional methods did well and were able to eliminate culturable bacteria, these methods often resulted in the development of “stunned” bacteria. The 3 heat-based methods (steam, boiling, microwave) all changed the mechanical properties of the catheters. Ultrasonic cleaning and vinegar showed evidence of viable but nonculturable bacteria populations, indicating the methods were bacteriostatic. Detergent and water wash followed by immersion in a commercially available 0.6% sodium hypochlorite solution and 16.5% sodium chloride (diluted Milton) gave consistent bactericidal results and no visible catheter damage.

While there is good agreement that intermittent catheterization (IC) represents the ideal method of managing a bladder that fails to empty, the optimal way to do IC is still a subject of controversy. The options around single use/multiple use, and the relative benefit of different types of catheter coatings are still debated. A recent, well done randomised trial in patients with spina bifida did not show a benefit in terms of UTI reduction over 8 weeks when intermittent catheters were reused compared to when catheters were only used once (35.2% vs 36.8%, p=0.877) (2). It is still common for patients to reuse uncoated catheters for IC (for example in Brazil, Canada and many developing countries), and thus the question about caring for reused catheters is still relevant.

Hydrophilic catheters have been introduced to the urological practice as an alternative to reduce the risk of recurrent UTI and urethral trauma (3). However, associated costs are higher in comparison to clean intermittent catheterization (CIC), which has been the standard since it was proposed by Lapides decades ago (4). One of the most frustrating things for patients is the lack of information from physicians about how to practically reuse catheters. In the neurogenic population, complex methods add an additional burden to a person’s daily routine. Over the years, patients have described varied routines, ranging from using one catheter a week and rinsing it in tap water, to more complex cleaning processes involving vinegar, dilute bleach, freezing and microwaving.

Despite the promising laboratory results published by Wilks et al, clinical studies are still needed to check whether diluted Milton strategy is effective and safe for patients. Bacterial biofilm still represents an important barrier for patients who reuse catheters (5). A general limitation of most uncoated PVC catheters is that they contain softeners such as phthalates that may put the users at risk of certain diseases (6). In the general population, exposure to some phthalate diesters is a cause of increasing concern because of their potential adverse effects on the reproductive and endocrine systems (7).
